# Isolated Celiac Artery Dissection Detected by Non-contrast CT

**DOI:** 10.7759/cureus.93734

**Published:** 2025-10-02

**Authors:** Motoyasu Nakamura, Keisuke Suzuki, Gen Inoue, Yuki Kaki, Akihito Kato, Kenta Watanabe, Hiroto Sasage, Kenji Dohi, Satoshi Suzuki

**Affiliations:** 1 General Medicine, Tone Health Cooperative Society, Tone Central Hospital, Gunma, JPN; 2 General Medicine, Kawasaki Health Cooperative Association, Kawasaki Kyodo Hospital, Kawasaki, JPN; 3 Emergency, Disaster and Critical Care Medicine, Showa Medical University School of Medicine, Tokyo, JPN

**Keywords:** abdominal pain, case report, celiac artery dissection, hypertension, non-contrast ct, smoking

## Abstract

Isolated celiac artery dissection (ICAD) is a rare condition commonly detected using contrast-enhanced CT (CE-CT). We report the case of a middle-aged Asian man with a history of hypertension, ureterolithiasis, and tobacco use who presented with persistent abdominal pain. Non-contrast CT (Canon Medical Systems Corporation, Japan) revealed celiac artery dilation and perivascular fat stranding, raising the suspicion of ICAD, which was subsequently confirmed by CE-CT. Conservative management was successful. This case highlights the potential value of non-contrast CT findings as early indicators of ICAD, emphasizing the need for clinical vigilance and timely CE-CT in high-risk patients.

## Introduction

Unlike aortic dissections, isolated celiac artery dissections (ICAD) without aortic involvement are rare [1]. Recent systematic reviews indicate that ICAD remains rare, accounting for approximately 0.1-0.2% of abdominal imaging findings. The majority of patients are male (73-88%) and middle-aged (mean age about 52 years), and major risk factors include hypertension (50-60%) and cigarette smoking (40-70%) [2]. The most common presenting symptom is acute or subacute epigastric pain (76%) or upper abdominal pain, but up to 18-20% of cases are asymptomatic and are found incidentally during imaging. Other symptoms may include nausea, vomiting, or back pain [3]. Differential diagnoses of ICAD include acute pancreatitis, gastrointestinal perforation, mesenteric artery dissection, atherosclerotic aneurysm, visceral artery aneurysms, and mesenteric ischemia, all of which can present with similar abdominal pain and imaging findings [4]. Most ICAD cases are managed conservatively with strict blood pressure control and monitoring, as symptoms typically resolve spontaneously. Endovascular treatment (e.g., stenting) or surgery is considered in less than 20% of cases, usually reserved for those with recurrent pain, signs of organ ischemia, progression of dissection, or aneurysmal rupture [5]. Reported complications include aneurysm formation, progression to arterial rupture, stenosis or occlusion of the celiac trunk, and consequent visceral organ ischemia. However, with timely diagnosis and appropriate management, major complications and mortality remain rare [6]. It has been detected in approximately 0.09% of abdominal contrast-enhanced CT (CE-CT) scans and 0.68% of abdominal CT scans performed for acute abdominal symptoms [7]. CE-CT is considered the gold standard for diagnosis, with the most common findings being an intimal flap, thrombosed false lumen, or aneurysmal dilatation [8]. Cases in which ICAD is suspected based solely on non-contrast CT findings are extremely rare. We report a case in which ICAD was initially suspected on non-contrast CT, which subsequently led to a definitive diagnosis on CE-CT.

## Case presentation

**Figure 1:**
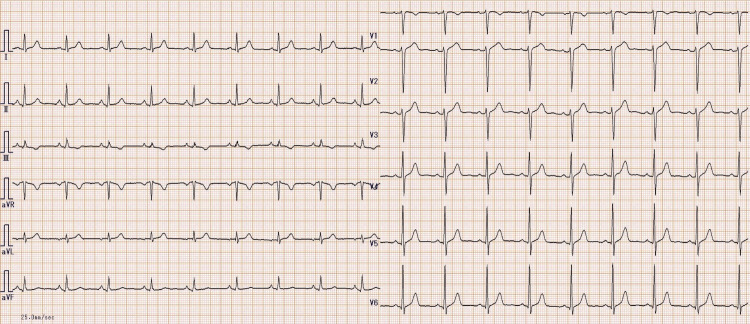
Electrocardiogram on admission Electrocardiogram showing sinus rhythm at 75 beats/min

A 42-year-old man with a history of hypertension and ureterolithiasis was referred to our hospital for further evaluation of persistent abdominal pain of approximately two weeks duration. The patient had no regular medications or known allergies and reported a smoking history of 16 pack-years. The patient denied alcohol consumption and had no relevant family history. On presentation, the vital signs were as follows: airway patent, respiratory rate 18 breaths/min, room air oxygen saturation of 100%, blood pressure 164/118 mmHg, heart rate 78 beats/min, Glasgow Coma Scale score of E4V5M6, and a body temperature reading of 37.1°C [9]. Physical examination revealed normal heart sounds and clear lung fields. Abdominal examination revealed a flat and soft abdomen with tenderness from the midline to the epigastric region without rebound. No edema, inguinal lymphadenopathy, or costovertebral angle tenderness was noted. Laboratory tests results showed mildly elevated levels of aspartate aminotransferase, alanine aminotransferase, g-glutamyl transferase, and C-reactive protein; D-dimer levels remained within the normal range (Table 1). Initial electrocardiogram (Nihon Kohden Corporation, Japan) showed a sinus rhythm at 75 beats/min (Figure 1).

**Table 1:** Laboratory data on admission WBC: White blood cell; RBC: Red blood cell; Hb: Hemoglobin; Plt: Platelets; TP: Total proteins; Amy: Amylase; Alb: Albumin; CRP: C-reactive protein; AST: Aspartate aminotransferase; Glu: Glucose; ALT: Alanine aminotransferase; PT: Prothrombin time; γ-GTP: Gamma-glutamyl transferase; CK: Creatine phosphokinase; T-bil: Total bilirubin; LDH: Lactate dehydrogenase; BUN: Blood urea nitrogen; Cre: Creatinine; Na: Sodium; K: Potassium; Cl: Chlorine; PT-INR: Prothrombin time/International Normalized Ratio; APTT: Activated partial thromboplastin time

Parameter	Measured Value	Reference Range	Units
WBC	9.9 × 10⁹	3.6–8.9 × 10⁹	/L
RBC	4.84 × 10¹²	4.3–5.7 × 10¹²	/L
Hb	15.9	13.1–16.6 (male)	g/dL
Plt	289 × 10⁹	140–340 × 10⁹	/L
TP	8.2	6.5–8.3	g/dL
Amy	54	37–132	U/L
Alb	4.7	4.1–5.1	g/dL
CRP	1.1	<0.3	mg/dL
AST	50	10–30	IU/L
Glu	94	70–110	mg/dL
ALT	78	10–30	IU/L
PT	170.4	70–130	%
γ-GTP	212	up to 50	IU/L
CK	108	60–250	IU/L
T-bil	0.85	0.2–1.2	mg/dL
LDH	686	124–222	IU/L
BUN	13.5	8–20	mg/dL
Cre	0.84	0.6–1.1	mg/dL
Na	139	135–145	mEq/L
K	5.5	3.6–5.0	mEq/L
Cl	103	98–108	mEq/L
PT-INR	0.76	0.9–1.1	——
APTT	29.3	24–36	s
D-dimer	0.2	<0.5	μg/mL
Hemolysis	3 +	Negative (“-”)	+/–
Urine-RBC	5–10 /field	0–3 /field	/HPF
Urine-WBC	1–2 /field	0–3 /field	/HPF
Urine-Cylinder	(+)	Negative	+/–
Urine-Bacteria	(–)	Negative	+/–

**Figure 2:**
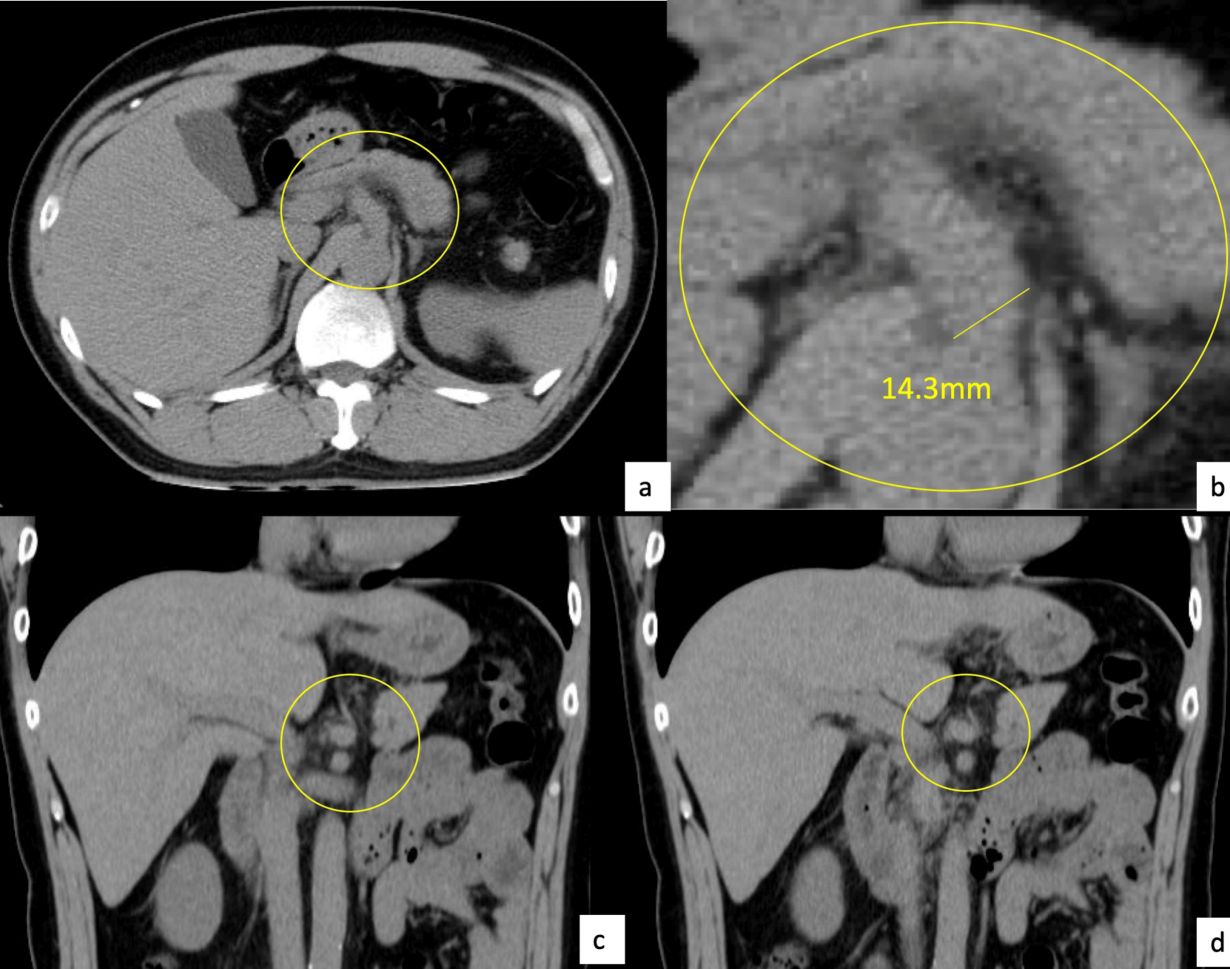
Abdominal non-contrast CT images Arterial dilatation of the celiac artery and opacification of the fatty tissue surrounding the celiac artery are observed (encircled)

Non-contrast abdominal CT (Canon Medical Systems Corporation, Japan) revealed celiac artery dilatation and perivascular fat stranding (Figure 2).

**Figure 3:**
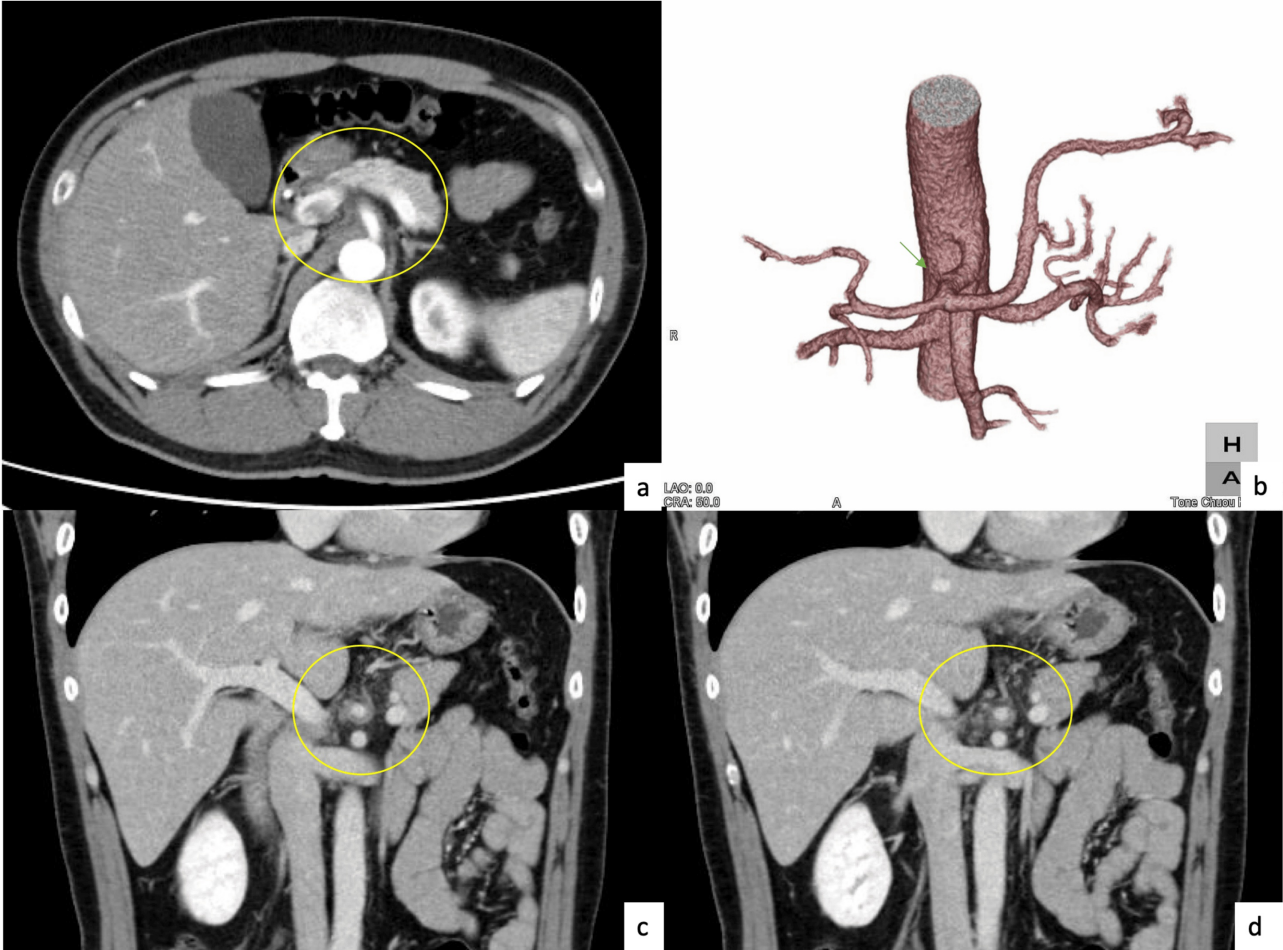
Abdominal CE-CT images False lumen occlusion-type celiac artery dissection (encircled) CE-CT: Contrast-enhanced CT

CE-CT revealed an ICAD with a thrombosed false lumen (Figure 3).

The patient was conservatively treated with blood pressure control and bed rest. On Day 2, follow-up laboratory data showed normalization of liver enzymes and C-reactive protein levels. Repeat CE-CT on Day 8 revealed no false lumen expansion or any evidence of organ ischemia. The patient was discharged uneventfully on day 10. No symptom exacerbations or further complications have been reported by the patient during outpatient follow-up for blood pressure management.

All medical equipment (Canon CT scanner, Nihon Kohden electrocardiogram machine) and assessment scales (e.g., Glasgow Coma Scale) used for diagnostic and monitoring purposes in this case are freely available for clinical and research purposes without license restrictions.

## Discussion

ICAD without an aortic dissection is rare. In the present case, a 42-year-old Asian man presented with persistent abdominal pain. Initial non-contrast abdominal CT revealed celiac artery dilation and perivascular fat stranding, raising suspicion of ICAD. ICAD predominantly affects middle-aged men (reported peak age of onset, 40 years) with cardiovascular risk factors, such as hypertension and smoking, and has a higher incidence in Asian populations than in Caucasian populations [10-12]. The typical diagnostic modality is CE-CT, which allows for identification of an intimal flap, patent or thrombosed false lumen, and aneurysmal dilatation [8,13,14]. In previous reports, such CE-CT findings led to early diagnosis and conservative treatment in the absence of organ ischemia [15]. The present case was consistent with the following characteristics: male sex, Asian ethnicity, fifth decade of life, heavy smoker, hypertension, and successful conservative management.

Although some studies have explored the role of ultrasound or magnetic resonance imaging for diagnosis, their diagnostic accuracy remains limited; thus, CE-CT continues to be the standard imaging modality [12,14]. Non-contrast CT is not typically considered a definitive diagnostic modality because of its low specificity. Findings such as perivascular fat stranding and arterial wall thickening can also be observed in malignant or inflammatory conditions, making them nonspecific [13]. Nevertheless, even without contrast enhancement, non-contrast CT can depict acute-phase changes that provide important diagnostic clues and may alert clinicians to the presence of serious underlying conditions, thereby contributing to early recognition and management. D’Ambrosio et al. demonstrated that mural thrombus, vessel expansion, and perivascular fat infiltration seen on non-contrast CT can raise suspicion for celiac artery dissection, prompting further investigation with CE-CT [4]. Similarly, Ichiba et al. reported that such secondary findings on unenhanced CT may guide clinicians toward early diagnosis in appropriate clinical scenarios [16]. These observations support our case, in which non-contrast CT served as a critical first step even before a definitive diagnosis was made by contrast imaging.

In one anatomical study, the mean diameter of the celiac artery was reported to be 7.9 ± 0.79 mm in women and 8.3 ± 1.08 mm in men [15]. In this case, the celiac artery diameter was 14.3 mm, supporting our suspicion of arterial dilatation even without contrast. Even when initial D-dimer levels were in normal range, likely due to the patient presenting 2 weeks after symptom onset-subacute phase-aligning with prior reports suggesting D-dimer’s reduced sensitivity as the disease progresses [17].

This case illustrates the diagnostic potential of non-contrast CT in detecting ICAD, particularly when classic features such as intimal flaps are not visible. When non-specific findings-such as celiac artery dilatation or perivascular fat stranding-are observed in high-risk patients, clinicians should maintain the suspicion of ICAD and promptly proceed to CE-CT. Given that non-contrast CT is often the first-line imaging modality in emergency settings, awareness of such secondary signs can prompt early diagnosis and management, potentially improving patient outcomes.

This report presents a single case, which limits the generalizability of the findings. Although CE-CT confirmed the diagnosis, the interpretation of non-contrast CT findings may be subject to bias, especially in retrospective studies. The absence of pathological confirmation limits the certainty of diagnosis. Additionally, the follow-up period was limited to a short hospital stay and long-term vascular outcomes were not assessed. Finally, further comparative studies are needed to establish diagnostic criteria or protocols for non-contrast CT in patients with suspected ICAD.

## Conclusions

In middle-aged male patients with a history of smoking who present with abdominal pain, clinicians should maintain a high suspicion index of ICAD, even when D-dimer levels are within the normal range. Non-contrast CT findings such as celiac artery dilation and perivascular fat stranding, although non-specific, may serve as important early indicators of ICAD. When these findings are present, CE-CT should be considered promptly to confirm the diagnosis. This case demonstrates that non-contrast CT may contribute to the early recognition of ICAD and highlights the importance of integrating subtle imaging findings with the clinical context in emergency settings.
